# Profile and prognosis of adolescents and adults with primary immunodeficiencies in the public health service in Brazil

**DOI:** 10.1186/1939-4551-8-S1-A134

**Published:** 2015-04-08

**Authors:** Fernanda Maia Vidal, Norma Rubini, Licia Clara Fernandes Couto Da Silva, Albertina Varandas Capelo, Maria Conceição Santos Moraes, Fernando Samuel Sion, Carlos Alberto Morais De Sa

**Affiliations:** 1 Federal University of the State of Rio De Janeiro, Brazil

## Background

Patients with primary immunodeficiencies (PIDs) are at high risk for potentially serious infectious and noninfectious complications. Early diagnosis and proper treatment enables better quality of life and survival. The aim of this study was to describe the profile and prognosis of adolescents and adults with PIDs in follow-up in a public hospital of Rio de Janeiro, Brazil.

## Methods

A retrospective study evaluating patients treated between 1997 and 2014 with a diagnosis of PID, aged > 12 years and in regular follow-up for > 1 year. The classification of the International Union of Immunological Societies Expert Committee for Primary Immunodeficiency in 2013 was used. Demographics, PIDs profile, time to diagnosis, treatment adherence and survival were analyzed.

## Results

33 patients were analyzed: 55% female, 70% Caucasian, 27% Black, and 3% Asian, with ages between 12 and 64 years (mean = 36.7, SD = 11) and length of follow-up between 1 and 17 years (mean = 8.84, SD = 11.29). The profile and frequency of PIDs was as follows: Predominantly antibody deficiencies (55%), Combined immunodeficiencies less profound than generally severe combined immunodeficiency (18%) Combined immunodeficiencies associated with features or syndromic (15%), Complement deficiencies (9%), and Autoinflammatory disorders (3%). The average time between onset of symptoms and diagnosis of PID ranged between 1 and 28 years (mean = 10.83, SD = 7.07). Compliance with treatment was good in 76% of patients, partial in 12% and 12% of treatment dropout. Survival between the onset of clinical manifestations and the end of the study ranged between 1 and 47 years (mean = 21.52, SD =13.79). Throughout the study period, one patient, with Wiskott-Aldrich syndrome and partial adherence to treatment, died due to septicemia and there were two deaths from cardiovascular disease.

## Conclusions

We observed that most patients were Caucasians, which differs from the racial distribution of the population of Rio de Janeiro, indicating the possibility of underdiagnosis in Black or lesser frequency of PID in this racial group population. Among the subgroups of PIDs, the predominantly antibody deficiencies occurred more often, which is in agreement with the international literature. A high percentage of patients showed good adherence to treatment, along with long-term survival. We found a great delay in diagnosis, which indicates the importance of training general practitioners and specialists for diagnostic screening of PIDs.

